# A Prospective, Randomized, Double-Blinded, Double-Dummy Pilot Study to Assess the Preemptive Effect of Triple Therapy with Aprepitant, Dexamethasone, and Promethazine versus Ondansetron, Dexamethasone and Promethazine on Reducing the Incidence of Postoperative Nausea and Vomiting Experienced by Patients Undergoing Craniotomy Under General Anesthesia

**DOI:** 10.3389/fmed.2016.00029

**Published:** 2016-07-05

**Authors:** Sergio Daniel Bergese, Erika G. Puente, Maria A. Antor, Adolfo L. Viloria, Vedat Yildiz, Nicolas Alexander Kumar, Alberto A. Uribe

**Affiliations:** ^1^Department of Anesthesiology, The Ohio State University Wexner Medical Center, Columbus, OH, USA; ^2^Department of Neurological Surgery, The Ohio State University Medical Center, Columbus, OH, USA; ^3^Department of Anesthesiology, Jackson Memorial Hospital, University of Miami, Miami, FL, USA; ^4^Center for Biostatistics, The Ohio State University, Columbus, OH, USA

**Keywords:** PONV, aprepitant, ondansetron, triple therapy, nausea, vomiting, craniotomy

## Abstract

**Introduction:**

Postoperative nausea and vomiting (PONV) is among the most common distressing complications of surgery under anesthesia. Previous studies have demonstrated that patients who undergo craniotomy have incidences of nausea and vomiting as high as 50–70%. The main purpose of this pilot study is to assess the incidence of PONV by using two different prophylactic regimens in subjects undergoing a craniotomy. Thus, we designed this study to assess the efficacy and safety of triple therapy with the combination of dexamethasone, promethazine, and aprepitant versus ondansetron to reduce the incidence of PONV in patients undergoing craniotomy.

**Materials and methods:**

This is a prospective, single center, two-armed, randomized, double-dummy, double-blind, pilot study. Subjects were randomly assigned to one of the two treatment groups. Subjects received 40 mg of aprepitant pill (or matching placebo pill) 30–60 min before induction of anesthesia and 4 mg of ondansetron IV (or 2 ml of placebo saline solution) at induction of anesthesia. In addition, all subjects received 25 mg of promethazine IV and 10 mg of dexamethasone IV at induction of anesthesia. Assessments of PONV commenced for the first 24 h after surgery and were subsequently assessed for up to 5 days.

**Results:**

The overall incidence of PONV during the first 24 h after surgery was 31.0% (*n* = 15) in the aprepitant group and 36.2% (*n* = 17) for the ondansetron group. The median times to first emetic and significant nausea episodes were 7.6 (2.9, 48.7) and 14.3 (4.4, 30.7) hours, respectively, for the aprepitant group and 6.0 (2.2, 29.5) and 9.6 (0.7, 35.2) hours, respectively, for the ondansetron group. There were no statistically significant differences between these groups. No adverse events directly related to study medications were found.

**Conclusion:**

This pilot study showed similar effectiveness when comparing the two PONV prophylaxis regimens. Our data showed that both treatments could be effective regimens to prevent PONV in patients undergoing craniotomy under general anesthesia. Future trials testing new PONV prophylaxis regimens in this surgical population should be performed to gain a better understanding of how to best provide prophylactic treatment.

## Introduction

Nausea and vomiting are known to be among the most common distressing postsurgical complications. Both of these conditions are associated with prolonged post-anesthesia care, delayed patient recovery and discharge, and increased overall cost of surgical intervention (1–7).

As stated in the guidelines from the Society for Ambulatory Anesthesia (SAMBA), postoperative nausea and vomiting (PONV) has been defined as the occurrence of nausea and vomiting during the first 24 h following a surgical procedure (1, 7).

Postoperative nausea and vomiting treatment and prevention may prove imperative to the postsurgical outcomes of patients undergoing craniotomies. For instance, retching and vomiting increases intracranial and intravascular pressure, leading to homeostatic changes and a decrease in cerebral perfusion (7). These factors are important to consider in neurosurgical settings as they can also increase the risk for brain swelling and surgical debridement following craniotomy (7). Furthermore, sedated patients are at higher risk of aspiration from vomiting due to the impairment of their airway reflexes; therefore, the use of antiemetic medications with few sedative properties are recommended as the preferred course of treatment (1, 8, 9).

Currently, multiple antiemetic medications are available for PONV prophylaxis. In various studies, regimens using single, double, and triple antiemetic agents have been proposed for PONV prophylaxis in patients undergoing craniotomies (1, 4, 10). However, an amalgam of regimens is the preferred prophylactic therapy, due to the additive antiemetic effects in various receptors of the emetogenic pathway (1, 11).

Alongside antiemetic medications, non-pharmacological techniques can be used as an alternative in preventing PONV. A commonly cited example is pericardium 6 acupoint stimulation, which reduces regurgitation and regulates gastric peristaltic movements (11). Despite mention of these strategies in the literature, these treatments have not yet been proven to be effective in preventing PONV (11).

One of the medications most commonly used for PONV prophylaxis is dexamethasone, an intravenous (IV) corticosteroid with anti-inflammatory properties, but with an unknown antiemetic mechanism of action (1, 8, 11). Data show that the use of dexamethasone enhances the post-discharge quality of recovery, in addition to reducing nausea, pain, and fatigue after surgery (8, 10, 12).

Promethazine is a phenothiazine with histamine (H_1_) antagonist activity. This time-tested antiemetic exults predominantly anti-dopaminergic effects as well as moderate anti-histaminic and anticholinergic properties. But, used at lower parental doses, promethazine has been reported to instigate highly sedative effects (3, 5, 13). Other risks are also attributed to promethazine. IV administration of promethazine may cause serious adverse reactions, including, but not limited to, contact dermatitis, inflammation, and venous thrombosis at the injection site (14). The drug also lowers the seizure threshold in patients with neurological deficits, characteristic of our patient population (15).

Ondansetron is a serotonin (5HT_3_) receptor antagonist and the most commonly studied drug for the prophylaxis of PONV in craniotomy procedures. It is typically considered the “gold standard” of PONV prophylaxis medications, due to its antiemetic properties and lack of sedative effects, and, as such, is widely used (1). These antiemetic and non-sedative qualities are particularly beneficial for neurological assessments following craniotomies, as patients remain more lucid.

Aprepitant is a selective antagonist with high affinity for the human neurokinin (NK_1_) receptor, which reduces the emetic effect of substance P and leads to increased oral bioavailability (2, 9, 10, 16, 17). The NK_1_ receptors are located within areas of the gut associated with the emetic reflex (17). As an antagonist, this drug plays an important role in decreasing substance P, which may have antiemetic effects both in the central and peripheral nervous systems (17).

Therefore, in this prospective, two-armed, randomized, double-blind, double-dummy trial, we hypothesized that using a triple therapy of aprepitant, promethazine, and dexamethasone versus a triple therapy of ondansetron, promethazine, and dexamethasone will effectively reduce the incidence of PONV in patients undergoing craniotomy.

## Materials and Methods

### Study Design and Patient Population

This study was designed as a randomized, double-blinded, doubled-dummy, single center, prospective pilot trial. After obtaining institutional review board (Office of Responsible Research Practices) approval for the research protocol, a total of 95 subjects provided their written informed consent before any study-related procedures began and completed the study at The Ohio State University Wexner Medical Center between January 2009 and April 2012. The clinical trial registry number of this study is NCT01474915.

Study inclusion criteria consisted of neurosurgical patients whose ages ranged from 18 to 85 years, with an American Society of Anesthesiologist (ASA) physical status of I, II, or III, scheduled to undergo elective craniotomy (opening of the cranium and dura mater) requiring at least 1 h of general anesthesia. These patients also had moderate-to-severe risk for PONV as assessed by having two or more risk factors on the simplified Apfel score. Exclusion criteria consisted of prisoners or mentally ill status, past medical history of alcohol or drug abuse, history of allergy reaction or intolerance to any study medications, pregnant or breastfeeding subjects, history of nausea and/or vomiting within 24 h of their procedure, history of treatment with antiemetic medication for nausea or vomiting within 24 h of their procedure, and history of chemotherapy treatment within 4 weeks prior to surgery. Only patients having craniotomy procedures and requiring admission were included. Patients who had received any medication with antiemetic properties prior to surgery were excluded from participating in the study.

The standardized anesthesia regimen also consisted of premedication of midazolam 1–2 mg IV directly before transferring the patient to the operating room. Anesthesia was induced with propofol 1–2 mg/kg IV and fentanyl 0.75–1.5 μg/kg IV. Tracheal intubation was performed after the administration of rocuronium 0.6–1.2 mg/kg IV. General anesthesia was maintained with volatile anesthetics (sevoflurane, desflurane, or isoflurane), and its titration concentration was guided on clinical judgment. Analgesia during anesthesia maintenance was provided with fentanyl boluses of 0.5–2.0 μg/kg IV. At the end of the procedure, neostigmine and glycopyrrolate were used to reverse residual neuromuscular block.

The method used for randomization was simple randomization using a random list generator. Consistent with a double-blind, double-dummy design, subjects were randomly assigned to one of the two treatment groups, Aprepitant or Ondansetron. Subjects received 40 mg of aprepitant pill (or matching placebo pill) 30–60 min before induction of anesthesia and 4 mg of ondansetron IV (or 2 ml of placebo saline solution) at induction of anesthesia. In addition, all subjects received 25 mg of promethazine IV and 10 mg of dexamethasone IV at induction of anesthesia. The health-care providers and researchers were blinded throughout the study, the only unblinded personnel was the pharmacist who prepared the study drug based on the randomization list.

Prior to surgery, vital signs and study safety procedures, including electrocardiogram (ECG), and urine or serum pregnancy tests were performed. Study medications were prepared by research pharmacists and administered during induction time (except aprepitant) by anesthesia care providers not involved in data collection. PONV assessments commenced after emergence from anesthesia and extubation time, and subjects were then transferred to the post-anesthesia care unit (PACU) or surgical intense care unit (SICU) when necessary.

### Outcome Measurement

This study explored the effects of triple therapy to prevent PONV as a primary endpoint, which is defined as nausea and/or vomiting during the first 24 h after a surgical procedure. Nausea was defined as any instance of feeling the urge to vomit and vomiting was defined as the ejection of gastric content through the mouth. Nausea was assessed by asking subjects to rate their nausea on a 0–10 point scale, with 0 being no nausea at all and 10 being severe nausea. Vomiting was assessed by asking subjects to rate their vomiting on a 0–3 point scale, with 0 being no vomiting, 1 being mild vomiting (1–2 episode in 12 h, small amount of emesis), 2 being moderate vomiting (3–5 episodes in 12 h, breakthrough vomiting), and 3 being severe vomiting (6–7 episodes in 12 h, intractable, incessant, projectile). After surgery, patients who experienced nausea and/or vomiting received ondansetron 4 mg IV as the initial rescue medication for PONV. Choice of subsequent rescue antiemetic was left to the anesthesiologist’s discretion. First episode of nausea, vomiting, and rescue medication was recorded.

Anesthesia and surgical procedure start and end time, admission and discharge time from the PACU/SICU, and general care floor were recorded. As a secondary outcome, the influences of these regimens were assessed from 24 h after surgery up to 5 days *via* direct subject interview and/or medical records review. Intraoperative medication and opioid daily consumption from PACU arrival time through a 5-day follow-up were collected. Subjects who were discharged before the end of a 5-day time period were contacted by telephone every 24 h to assess nausea and/or vomiting, rescue medication, opioid consumption, and adverse events and serious adverse events. Following the first 24 h after administration of the prophylactic triple therapy, an ECG was performed as part of safety assessments.

### Statistical Analysis

A previous study published in 2005 by Gan et al. compared the use of pre-operative dose of aprepitant 40 mg orally to ondansetron 4 mg IV for prevention of PONV; the investigators reported that only 15% of the patients experienced vomiting in aprepitant group compared with 33% vomiting incidence in ondansetron group (18). In a two-sided test to compare the two proportions at 0.05 significance level, *n* = 88 subjects per group were assigned in order to achieve 80% power.

However, after accruing more than 50% of the study participants, the overall PONV incidence was greater than anticipated; thus, the investigators decided to run an unplanned data analysis. The results of this unanticipated analysis showed only a 5.2% absolute difference in PONV (ondansetron group 36.2% versus aprepitant group 31%) between both groups, indicating that our estimation of sample size was not realistic. To detect such a small difference in proportion, an overall simple size of 2590 patients (1295 per group) was required. Due to lack of statistical significance differences, we decided to stop the enrollment, and the available data are the subject of this publication. Descriptive statistics are reported as mean ± SD, median (range), total number, and percentage. The data were analyzed using Statistical Analysis Software, version 9.3 (SAS Institute Inc., Cary, NC, USA). Significance was accepted if *P* ≤ 0.05.

## Results

**Figure 1:**
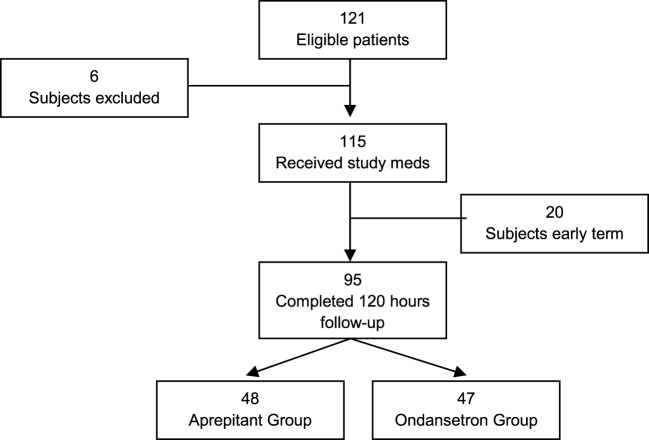
**Patient screening flowchart**.

A total of 121 subjects were screened and enrolled to participate in this study. However, 6 subjects were considered screen failures due to failure to meet inclusion criteria or meeting exclusion criteria, and 20 subjects were removed from the study due to protocol deviations that could affect our data analysis. Deviations from the protocol included anesthesia care staff not administrating the triple therapy as directed, prolonged intubation after surgery, and/or loss of follow-up. Ninety-five subjects completed the study: 48 in the aprepitant group and 47 in the ondansetron group (Figure 1). No adverse events related to study medications were documented.

There were no statistically significant differences among subjects’ demographics, risk factors for PONV, duration of anesthesia, and postoperative opioid consumption between the two groups (Table 1).

**Table 1:** **Patient demographics and surgical variables**.

Demographics and surgical variables	Aprepitant group	Ondansetron group
Number of subjects	48	47
Age, mean (SD), years	52.1 ± 14.5	51.4 ± 16.8
Weight, mean (SD), kg	86.2 ± 21.6	86.8 ± 18.6
Height, mean (SD), cm	170.7 ± 10.3	169.3 ± 10.5
BMI, mean (SD)	29.5 ± 6.5	30.5 ± 7.3
Race-White, *n* (%)	46 (96)	43 (91)
ASA I/II/III	13/34/1	13/28/6
Female, *n* (%)	26 (54)	25 (53)
History of PONV and/or motion of sickness *n* (%)	19 (40)	17 (36)
Non-smoker status, *n* (%)	35 (73)	36 (77)
Postoperative opioids, *n* (%)	48 (100)	47 (100)
Apfel risk factors, *n* (%)		
1 (Low risk)	12 (25)	11 (23)
2 (Moderate risk)	19 (40)	21 (45)
3 or 4 (High risk)	17 (35.4)	15 (32)
Duration of anesthesia, mean (SD), h	5.14 ± 2.20	5.75 ± 3.15
Duration of SICU stay, mean (SD), h	35.6 ± 27.3	45.0 ± 37.7
Duration of total hospital stay, mean (SD), h	53.3 ± 62.0	55.0 ± 48.6

*n, total number; %, percentage*.

The overall incidence of PONV during the first 24 h after surgery was 31.0% (*n* = 15) in the aprepitant group and 36.2% (*n* = 17) for the ondansetron group.

The median severity of postoperative nausea between the aprepitant group and the ondansetron group was 6 and 5, respectively (Table 2). The overall incidence of vomiting during the first 24 h in the aprepitant group was 8% (*n* = 4), with a median worst vomiting score of 2.5. Similarly, the occurrence and severity of postoperative vomiting in the ondansetron group was 12.8% (*n* = 6), with a median worst vomiting score of 3. Rescue antiemetics during the first 24 h after surgery were required for 29% (*n* = 14) of the aprepitant group and for 36.2% (*n* = 17) of the ondansetron group. There were no statistically significant differences between PONV outcome variables between the two groups (Table 2).

**Table 2:** **Postoperative nausea and vomiting outcome variables in the first 24 h**.

	Aprepitant group	Ondansetron group	*P*-value
Number of subjects	48	47	NA
PONV, *n* (%)	15 (31)	17 (36.2)	0.611
Vomiting, *n* (%)	4 (8)	6 (12.8)	0.481
Worst vomiting score, median (IQR)	1.5 (1, 2.5)	3 (3, 3)	0.100
Any nausea incidence, *n* (%)	15 (31)	17 (36.2)	0.611
Significant nausea incidence (A score ≥4 on the VRS), *n* (%)	11 (23)	14 (29.8)	0.445
Worst nausea score, median (IQR)	6 (3, 7)	5 (4, 7)	0.705
Rescue antiemetics, *n* (%)	21 (44)	25 (53)	0.357
Postoperative opioid consumption (oral morphine mg), median (IQR)	54.0 (37.5, 105.0)	75.0 (30.0, 122.5)	0.830

*IQR, interquartile range; %, percentage; n, total number; NA, not applicable; VRS, verbal rating scale*.

From the period of 0 to 120 h following surgery, the severity of vomiting was significantly lower in the aprepitant group compared with the ondansetron group (2 versus 6%, *P* = 0.008). However, there was no difference between the two groups from 0 to 2, 0 to 24, or 24 to 120 h (Table 3).

**Table 3:** **Nausea and vomiting outcome variables (0–120 h)**.

Outcome variables	0–2 h		24–48 h		24–72 h		24–96 h		24–120 h	
	Aprepitant	Ondansetron	Aprepitant	Ondansetron	Aprepitant	Ondansetron	Aprepitant	Ondansetron	Aprepitant	Ondansetron
Number of subjects	48	47	48	47	48	47	48	47	48	47
PONV, *n* (%)	15 (31.3)	17 (36.2)	13 (27)	8 (17)	16 (33)	8 (17)	16 (33)	16 (34)	17 (35)	16 (34)
Vomiting, *n* (%)	4 (8.3)	6 (12.8)	1 (2.1)	4 (8.5)	2 (4.2)	4 (8.5)	2 (4.2)	4 (8.5)	3 (6.3)	4 (8.5)
Worst vomiting score, median (IQR)	1.5 (1, 2)	3 (3, 3)	1 (1, 1)	2 (2, 2.5)	1.5 (1, 2)	2 (2, 3)	1.5 (1, 2)	2.5 (2, 3)	1 (1, 2)	2.5 (2. 3)
Any nausea incidence, *n* (%)	15 (31.3)	17 (36.2)	12 (25)	8 (17)	15 (31.3)	8 (17)	15 (31.3)	16 (34)	16 (33.3)	16 (34)
Significant nausea incidence (A score ≥4 on the VRS), *n* (%)	3 (10)	7 (23)	11 (22.9)	7 (14.9)	16 (17)	9 (10)	21 (15)	15 (11)	25 (13)	17 (9)
Worst nausea score, median (IQR)	6 (3, 5.8)	7 (5, 10)	6 (5, 8)	7 (6, 7.5)	6.5 (4.5, 8)	7 (6, 9)	7 (5, 8)	6 (4, 7)	6 (4, 8)	7 (6, 9)
Rescue antiemetics, *n* (%)	3 (6)	9 (19)	5 (10)	3 (6)	6 (12.5)	3 (6)	6 (12.5)	7 (15)	7 (15)	8 (17)
Postoperative opioid consumption (oral morphine mg), median (IQR)	NA	NA	41.2 (15.0, 103.5)	46.2 (15.0, 84.0)	45.0 (15.0, 105.0)	38.7 (15.0, 69.0)	45.0 (15.0, 92.5)	40.0 (15.0, 69.0)	40.0 (15.0, 90.0)	38.7 (18.0, 72.0)

*VRS, verbal rating scale; IQR, interquartile range; %, percentage; 0 hour, End of Surgery (closure time)*.

There were no statistically significant differences between the two groups in the number of vomiting episodes, incidence or severity of nausea, need for rescue antiemetic’s, or complete response from 0 to 2, 0 to 24, 0 to 120, or 24 to 120 h (Tables 2 and 3).

The median time to first emetic and significant nausea episodes was 7.6 (2.9, 48.7) and 14.3 (4.4, 30.7) hours, respectively, for the aprepitant group and 6.0 (2.2, 29.5) and 9.6 (0.7, 35.2) hours, respectively, for the ondansetron group. The median time for the first rescue medication was 15.3 (5.3, 31.2) hours for the aprepitant group and 9.6 (0.7, 35.2) hours for the ondansetron group. There were no statistically significant differences in these times (Table 4).

**Table 4:** **Intent to treat population**.

Time to treatment failure, median (IQR), h	Aprepitant group, *N* = 48	Ondansetron group, *N* = 49	*P*-value
Time to first emetic episode	7.6 (2.94, 48.7)	6.0 (2.2, 29.5)	0.483
Time to first rescue	15.3 (5.3, 31.2)	9.6 (0.7, 35.2)	0.444
Time to first significant nausea	14.3 (4.4, 30.7)	9.6 (0.7, 35.2)	0.444

*IQR, interquartile range; N, total number*.

## Discussion

This pilot study presents similar results between the triple therapy of aprepitant, promethazine, and dexamethasone versus the triple therapy of ondansetron, promethazine, and dexamethasone in preventing nausea and vomiting after craniotomies under general anesthesia. Therefore, both treatments could be considered adequate alternatives for PONV treatment when compared with other treatment regimens in subjects undergoing craniotomy. On the one hand, the results demonstrate no statistical significance between both groups. On the other hand, when compared with the overall incidence of PONV in general population (30–80%) and patients undergoing craniotomy (43–70%), both treatments lowered the incidence of PONV in all subjects, and even more significantly in subjects with three or four of Apfel’s risk factors (1, 6–8, 12).

Postoperative nausea and vomiting is classed as early PONV (on-setting between 0 and 2 h after surgery) or delayed PONV (on-setting between 2 and 24 h after surgery) (1, 7). Additionally, episodes of nausea and vomiting that occur after the patient leaves an institution is classified as post-discharge nausea and vomiting (PDNV) (1, 7). Several studies have reported a general postoperative incidence of 30% for nausea and 50% for vomiting, and as high as 80% incidence in patients classified as high risk by the Apfel Simplified Risk Score (1, 6–8, 12).

Current guidelines from SAMBA that include data from several meta-analyses confirm that the known risk factors for PONV do indeed factor into a patient’s development of PONV (1). As a result, the Apfel Score continues to be the preferred pre-operative tool for PONV assessment (1, 19). The simplified Apfel score for the prediction of PONV is based on the following four criteria: female gender, history of PONV or a past medical history of motion sickness, non-smoking status, and expected postoperative opioid use. Each risk factor will increase the incidence of PONV in patients by ~20%, female gender being the strongest predictor (1, 19). The Apfel score considers low, medium, and high risk of developing PONV to be patients with 0–1, 2 or 3, and more than 3 risk factors, respectively (19). Consequently, it has been reported that patients presenting two or more risk factors are more likely to benefit from treatment prophylaxis. Also, the current SAMBA guidelines showed that PONV incidences were higher in cholecystectomy and gynecology surgeries (1, 20); nevertheless, previous studies demonstrated that patients who have undergone craniotomies had an average incidence of PONV as high as 50–70%. However, most of these studies did not clearly document standardized treatment regimens (1, 4, 7, 10, 11).

The use of a combination of two or more drugs to prevent PONV is known as multimodal therapy, and its use is recommended in patients with high risk for PONV (1). The multimodal approach recommended by current SAMBA guidelines in adults includes double therapy with 5-HT3 receptor antagonist + dexamethasone or droperidol, dexamethasone + droperidol or ondansetron + casopitan or triple therapy with 5-HT3 receptor antagonist + dexamethasone + droperidol (1). Conversely, there are not sufficient studies reporting the experience of using multimodal approach with double or triple therapy, particularly in the craniotomy patient population (Table 5). Therefore, the need for multimodal therapy trials with different regimens to prevent PONV was necessary.

**Table 5:** **Characteristics of studies included in the discussion**.

No.	Reference	Surgery type	Intervention	No. of groups	Measured outcome and time	Study design	Results
1	Gan et al. (2)	Open-abdominal surgery under general anesthesia	PO aprepitant 40 mg or PO aprepitant 120 mg or IV ondansetron 4 mg	3	Incidence of vomiting Need for rescue Nausea score Complete response Cumulative incidence for day 1	Multicenter double-blind randomized trial	Complete response was not different between groups Aprepitant groups experienced lower incidence of postoperative vomiting than ondansetron group No significant difference in nausea score and use of rescue between groups No significant differences between the aprepitant groups
2	Tsutsumi and Kakuta (9)	Elective craniotomy under general anesthesia	IV fosaprepitant 150 mg or IV ondansetron 4 mg	2	Incidence of PONV Need for rescue Pain score Cumulative incidence until day 2 with individual values for day 1	Prospective double-blind randomized trial	Fosaprepitant group experienced lower incidence of vomiting than ondansetron group Incidence of complete response higher in fosaprepitant group than ondansetron group
3	Habib et al. (10)	Craniotomy under general anesthesia	PO aprepitant 40 mg or IV ondansetron 4 mg	2	Incidence of vomiting Need for rescue Nausea score Cumulative incidence until day 2 with individual values for day 1	Prospective double-blind randomized trial	Aprepitant group experienced lower incidence of postoperative vomiting Nausea score and need for rescue were not different between groups
4	Rapoport et al. (17)	Moderately emetogenic chemotherapy	PO aprepitant 125 mg, PO ondansetron 8 mg, PO dexamethasone 12 mg prior to chemo, and PO aprepitant 80 mg for 2 days post-chemo or PO ondansetron 8 mg, PO dexamethasone 20 mg prior to chemo and PO ondansetron 8 mg for 2 days post-chemo every 12 h	2	Incidence of vomiting Complete response Cumulative incidence until day 5	Prospective double-blind randomized parallel-group trial	Aprepitant group experienced significantly lower incidence of vomiting for 2 days post-chemo
5	Long et al. (21)	Elective hysterectomy	PO aprepitant 40 mg or placebo	2	Incidence of vomiting Need for rescue Nausea score Cumulative incidence for day 1	Prospective double-blind randomized trial	Trend suggesting reduction in vomiting, nausea score, and need for rescue in aprepitant group compared with placebo group
6	Diemunsch et al. (22)	Open-abdominal surgery under general anesthesia	PO aprepitant 40 mg or PO aprepitant 125 mg or IV ondansetron 4 mg	3	Incidence of vomiting Need for rescue Nausea score Complete response Cumulative incidence until day 2 with individual values for day 1	Double-blind randomized phase III trial	Aprepitant groups non-inferior to ondansetron group for complete response Aprepitant groups superior to ondansetron group in preventing vomiting up to 48 h postoperatively Distribution of peak nausea scores lower in aprepitant groups compared with ondansetron group Similar efficacy between the two aprepitant groups, suggesting a possible plateau in response
7	Ham et al. (23)	Laparoscopic gynecologic surgery	IV 4 mg Ondansetron in combination with PO aprepitant 80 mg or placebo	2	Incidence of PONV Need for rescue Impact of PONV on daily life by FLIE Cumulative incidence until day 2	Prospective double-blind randomized trial	Incidence of PONV within an hour postoperatively was lower in aprepitant group than in placebo group No difference in use of rescue between groups No difference in impact of PONV on daily life between groups
8	Tsutsumi et al. (24)	Craniotomy under general anesthesia	IV 150 mg fosaprepitant or IV 4 mg ondansetron	2	Incidence of PONV Need for rescue Pain score Cumulative incidence until day 2	Prospective double-blind randomized trial	Percentage of patients who experienced vomiting from 0 to 24 h and 0 to 48 h in the fosaprepitant group was lower than in the ondansetron group Complete response rate from 0 to 24 h and 0 to 48 h was greater in the fosaprepitant group than in the ondansetron group No significant difference in incidence of PONV or need for rescue between the groups
9	Shilpa et al. (25)	Thyroidectomy	PO ondansetron 8 mg or PO clonidine 150 μg	2	Incidence of PONV Incidence of sore throat Cumulative incidence for day 1	Prospective double-blind randomized trial	A smaller proportion of patients in the ondansetron group developed PONV than did patients in the clonidine group Moderate sore throat development was the same in both groups
10	Alonso-Damián and Anguiano-García (26)	Open cholecystectomy under general anesthesia	PO aprepitant 80 mg or IV ondansetron 4 mg	2	Incidence of PONV Cumulative incidence for day 1	Prospective double-blind randomized trial	Aprepitant group experienced less postoperative nausea and vomiting than ondansetron group

*PO, postoperative; IV, intravenous; PONV, postoperative nausea and vomiting; FLIE, Functional Living Index-Emesis*.

A randomized trial conducted by Habib et al. compared the use of aprepitant and dexamethasone versus ondansetron and dexamethasone to prevent PONV in patients undergoing craniotomy (10). Based on data analysis, 104 subjects completed the study. Of these, 53 subjects were allocated to the ondansetron group and 51 to the aprepitant group. The study concluded that the incidence of vomiting during the early postoperative period (0–2 h) was significantly lower in the aprepitant group versus the ondansetron group (6 versus 21%) (10). In addition, the authors reported 36% incidence of vomiting for the ondansetron group and 14% for the aprepitant group. However, there was no statistical difference in the incidence and severity of nausea between both groups during the early and/or 24 h postoperative period (10).

The literature suggests that the impact of NK_1_ antagonists, such as aprepitant, on the central nervous system may possess a better efficacy than 5HT_3_ receptor antagonists, which have a mechanism of action predominately on the peripheral level (2, 9, 10, 16, 17). NK_1_ antagonists have also demonstrated their efficacy in the prevention of chemotherapy-induced nausea and vomiting in patients receiving moderately emetogenic chemotherapy (17). Hence, it has also been proposed to be an effective PONV prophylactic medication in neurosurgical procedures. Aprepitant has been documented to be effective up to 48 h postoperatively, with minimal sedative effects (2, 16).

Gan et al. published a pilot study exploring the role of aprepitant in preventing PONV. In this double-blinded study, 805 patients undergoing open-abdominal surgery were randomly assigned a treatment of 40 mg aprepitant orally, 125 mg aprepitant orally, or 4 mg ondansetron IV in order to evaluate PONV and necessity for rescue therapy up to 48 h postoperatively (2). Aprepitant proved to be superior to ondansetron for the prevention of postoperative vomiting (90% with 40 mg aprepitant, 95% with 125 mg aprepitant, and 74% with ondansetron) (2). No significant differences in PONV were found between the two doses of aprepitant (2). Also, no significant differences were noted between aprepitant and ondansetron for nausea control or the need for rescue (2).

Long et al. performed a prospective, randomized, placebo controlled outcomes trial of aprepitant with patients undergoing elective hysterectomy (21). This study catered toward a population where Apfel’s risk factors were considered (21). A total of 256 women received either oral aprepitant 40 mg or placebo in addition to an established prophylactic regimen of dexamethasone and ondansetron (21). There was a trend showing that the addition of aprepitant to the regimen lowered the incidence of both nausea (24 versus 38%) and vomiting (17 versus 29%) over the first 24 h after surgery compared with the placebo (21). The need for additional antiemetic medication was also lower in the aprepitant group than in the placebo group (42 versus 60%) (21).

In 2007, Diemunsch et al. explored aprepitant’s NK_1_ antagonist properties as a preventative measure for PONV in patients undergoing open-abdominal surgery (22). They compared the efficacy of IV ondansetron 4 mg to experimental doses of oral aprepitant 40 mg and oral aprepitant 125 mg. Both experimental doses of aprepitant were shown to be no less effective in achieving the study’s primary endpoint – that is, no vomiting or use of rescue therapy 0–24 h after surgery (CI > 0.65) (22). Additionally, it was shown that aprepitant was greatly more effective in preventing vomiting and reducing nausea from 24–48 h after surgery (22).

Ham et al. performed a similarly designed prospective study that investigated the effects of combining aprepitant and ondansetron in patients at high risk for PONV up to 48 h postoperatively in female patients undergoing laparoscopic gynecologic surgery (23). Patients were randomly assigned to receive IV ondansetron 4 mg and oral aprepitant 80 mg or IV ondansetron 4 mg and a placebo (23). When examining a smaller time interval, 0–1 h, there was no significant difference between the incidences of PONV (23). However, the aprepitant–ondansetron therapy proved to be more effective in reducing PONV incidence 1–48 h after surgery compared with ondansetron alone (8 versus 19%) (23).

Tsutsumi et al. designed a similar randomized study that evaluated the effects of IV fosaprepitant versus IV ondansetron on the prevention of PONV in neurosurgery patients (24). Fosaprepitant is a chemically modified form of aprepitant that increases its solubility and that, once delivered into the body, is converted to aprepitant (24). When examining smaller time intervals, 0–2 h, there were no significant differences between the incidences of PONV (24). However, the fosaprepitant group demonstrated a higher complete response ratio as well as a lower incidence of vomiting when compared with ondansetron at the 24- and 48-h time point (24). Their results further suggest that the use of NK_1_ antagonists may be advantageous in diminishing episodes of vomiting if given pre-operatively in this type of surgery (24). Additionally, the use of IV fosaprepitant could substitute oral aprepitant because it satisfies anesthetists’ concerns about no oral consumption before surgery while still displaying the effective antiemetic effects of aprepitant.

Shilpa et al. published a double-blind, randomized controlled trial comparing the efficacy of clonidine versus ondansetron (25). This prospective study randomly assigned 60 patients with an ASA score of I or II into an ondansetron group or a clonidine group (25). Those in the ondansetron group received 8 mg of oral ondansetron, while the clonidine group received 150 μg of clonidine as premedication. Both groups received 8 mg of dexamethasone intravenously. A larger percentage of patients in the clonidine group developed PONV than in the ondansetron group (36.7 versus 30%) (25). For the patients who experienced nausea and vomiting, those in the clonidine group experienced it during the 1–2 h postoperative period and those in the ondansetron during the 6–12 h postoperative period (25). Therefore, the study concluded that ondansetron with dexamethasone is more effective in controlling PONV when compared with clonidine with dexamethasone (25). Nonetheless, in our study, 36.2% of the patients in the ondansetron group experienced PONV.

In 2012, Alonso-Damián and Anguiano-García compared the efficacy of aprepitant 80 mg and ondansetron 4 mg in preventing PONV in a total of 60 patients undergoing open-abdominal surgery (26). The study’s results showed that subjects who had been given aprepitant had less nausea than the subjects who had been dosed with ondansetron on arrival to the post-anesthesia recovery room (3.3 versus 53.3%; *P* < 0.001) and 6 h postoperatively (0 versus 33.3%; *P* = 0.002) (26).

A meta-analysis of three studies (Gan et al., Diemunsch et al., and Habib et al.) with 1171 total subjects, performed by Liu et al., compared the efficacy of aprepitant 40 mg and ondansetron 4 mg in preventing PONV. The meta-analysis demonstrated that the dose of aprepitant was more effective than the dose of ondansetron in preventing vomiting (*P* < 0.001) (27). The overall incidence of PONV for subjects dosed with aprepitant was 13.3% (95% CI = 9.5–18.4) and 28.4% (95% CI = 24.6–32.9) for subjects dosed with ondansetron (27).

A recently published, evidence-based review by Milnes et al. evaluated the efficacy of aprepitant in decreasing the incidence of PONV (28). After a thorough search using CINAHL, MEDLINE, EMBASE, and Google Scholar for publications dating from 2007 to 2014, 23 articles were found, 10 of which were relevant to our study (28). Eight randomized controlled trials, one prognostic study, and one *post hoc* analysis were reviewed (2, 10, 22, 28–34). Although the end points of the studies varied, all of the studies concluded that aprepitant decreased the incidence of PONV as compared with ondansetron (28).

In regard to its approved role as an add-on to standard antiemetic treatment, aprepitant holds promising evidence of clinical effectiveness. Nevertheless, aprepitant’s high price raises the question of its cost-effectiveness (35). Utilizing aprepitant in 100 patients costs $31,000 (25,000€), while aprepitant’s average clinical benefit is the avoidance of 15 cases of PONV for every 100 patients (35).

Our study had some limitations that should be recognized. The first limitation is the anesthesia care provider’s concern of the pre-operative oral administration of aprepitant/placebo. As a result, standard anesthesiologists’ suggestion of 12 h of pre-operative fasting was not met. A future trial should experiment with fosaprepitant, which is delivered into the body intravenously and then converted to aprepitant (24). Another limitation that should be addressed is that postoperative opioids were administered to all patients as needed, despite introducing them to an additional Apfel risk factor for PONV. As a result, patients who reported experiencing more pain required higher doses of rescue opioid medications. Because patients received variable doses of postoperative opioids, a confounding variable was introduced into this study, as higher doses of opioids further increase the risk for PONV. Also, the type of surgery that patients in the study underwent may also have interfered in the results of our study. The postoperative symptoms of subjects undergoing craniotomies include, but are not limited to, headaches, dizziness, and nausea. Due to the nature of this type of surgery, it was impossible for us to associate these symptoms specifically to the surgical procedure, anesthesia regimen, or adverse events related to study medications. Another limitation of our study was a discrepancy between the approved and our standard of care administration time of ondansetron IV. The packaging of ondansetron instructs anesthesia care providers to administer it at induction of anesthesia. Nevertheless, in a clinical setting, they administer the drug at the end of surgery, right before anesthesia emergence. On the other hand, the literature shows that antiemetics are more effective when administered toward the end of surgery rather than induction (16). For instance, it takes 3 h for aprepitant to reach its maximum blood concentration; therefore, administrating it at the end of surgery could reduce the optimal capacity of its antiemetic properties after anesthesia emergence. And finally, the recommended rescue therapy for both groups was the administration of ondansetron 4 mg IV. The study design intended to apply standard of care institutional guidelines in order to maintain consistency of rescue medication administration. It might be arguable that patients randomized to the ondansetron arm received, as a rescue medication, the same drug, leading to double dosing. Future trials will benefit from a detailed analysis of our own pilot study limitations.

## Conclusion

The primary goal of this pilot study was to compare the efficacy of triple therapy with aprepitant, dexamethasone, and promethazine versus triple therapy with ondansetron, dexamethasone, and promethazine for the prevention of PONV in patients undergoing craniotomies. Our results showed an overall incidence of PONV of 31.0 and 36.2% for aprepitant and ondansetron, respectively. In conclusion, this study showed similar adequacy of both PONV prophylaxis regimens in subjects undergoing neurological surgery with general anesthesia. Future trials testing new PONV prophylaxis regimens in this surgical population should be performed to gain a better understanding of how to best provide prophylactic treatment.

## Author Contributions

SB and EP designed the study, developed the methodology, and wrote the manuscript. AV, AU, and MA developed the methodology, collected the data, performed the analysis, and wrote the manuscript. NK and VY performed the analysis and wrote the manuscript.

## Conflict of Interest Statement

The authors declare that the research was conducted in the absence of any commercial or financial relationships that could be construed as a potential conflict of interest.
